# Treatment of Scrofulous Lymphatic Glands

**Published:** 1861-01

**Authors:** P. C. Price


### TREATMENT OF SCROFULOUS LYMPHATIC GLANDS.

BY P. C. PRICE, ESQ., &c.

General Treatment.—In all diseases in which complication exists, prescribed forms of treatment must be more or less modified; and this observation is particularly relevant as regards the therapeutical management of this special glandular affection. It has been stated that, in all probability, in the majority of cases in which this derangement occurs, it obtains as a result of certain causes which have an extra lymphatic origin; and, therefore, before any hopeful treatment can be adopted, it is highly advisable that they should, if possible, be recognized. What these causes may be, it is often difficult to determine; but frequently a decided departure from health will point to some impairment of the digestive, secretive, or absorptive functions. It must be allowed, I think, that this kind of glandular affection is generally met with in each of the divisions which so commonly admit the development of strumous disease, viz.: the sanguine, phlegmatic, lymphatic; so that if in certain instances it appears to be but slightly, if at all associated with well developed scrofula, still it must be ordinarily considered as in some way or other connected with this peculiar diathesis. Believing that the simpler forms of the affection, when appearing in the external glands, although not appreciable in those of the mesentry and other internal structures, are dependent on systematic causes, I usually commence the treatment by regulating the proper action of the various viscera. This is best accomplished when the *primæ viæ* are at fault, by unloading the bowels, and maintaining their proper action. Constipation is a very frequent accompaniment of glandular swellings, and it is astonishing to see how an alterative course of medicines is attended with almost immediate good effect, provided the disease has not been too long established. Mr. Abernethy was in the habit of treating these cases with calomel; but with what special views he used this powerful medicine does not clearly appear even from his own description. Although as a rule I do not resort to the administration of calomel in large and continuous doses, still there is no doubt that mild mercurial preparations are often of value, as the liver is generally torpid in its functions. For reasons already stated, I prefer small and repeated exhibitions of hydrargyrum cum creta in combination with compound rhubarb powder, or some alterative. To improve the tone of the digestive and assimilative powers is all important; for, as long as these remain neglected, the proper action of the various organs cannot take place. Mineral acids in combination with bark, quinine and iron are the most available adjuncts for restoring a healthy tone. For the more immediate treatment —reduction—of the disease, iodine, bromine and cod-liver oil have, during the past quarter of a century, taken the first rank; and, if cure is to be obtained, perhaps no specific remedies are more valuable. Iodine and bromine may be exhibited in one or more of the forms already described, or in any of those which will meet with further consideration. When speaking of the action of iodine on indurated glands from vascular derangement, it was stated that the administration of the drug was not always so rapid or certain in its effects as could be wished for; and that not unfrequently disappointment followed even its most pertinacious use. This remark is even more applicable to the treatment by iodine of this form of glandular mischief than the last described; for considerable experience has proved to me that even its very extended exhibition is oftentimes attended with failure. Various reasons may be assigned for this want of success: but, whatever may be the correct one, it is more than probable that in the majority of cases of confirmed mischief a hidden influence is at fault in keeping up the irritation, over which iodine has little or no control. In one instance, in particular, in which the constitution was saturated with iodine, little or no amendment or reduction in the gland swellings resulted. It may be asked—this important agent being oftentimes incapable of inducing resolution, or even arresting the further progress of the disease—what means should be adopted to procure amendment and cure? I fear even the most sanguine and experienced practitioner will readily admit that many cases, in which this form of glandular affection exists, are not really amenable to the treatment of any fixed medicinal means, and that the good to be expected is chiefly derivable from change of climate, and from the removal of those unwholesome influences to which the subject of the affection may be exposed. Rapid amendment in such cases, occurring especially among the poor, by this plan of rational treatment is frequently most marked. Much has been said and written on the direct advantage of sea-air and sea-bathing; and, while many excellent writers have been far too laudatory in their recommendation of these means, others have not sufficiently noticed the benefits which their employment often affords. Ample observation among both private and hospital patients at Margate, has assured me that immediate advantage is very frequently to be gained by a sea-side residence, and a free recourse to sea-bathing, provided it be not contra-indicated by reason of certain complications of constitution and disease. If, with these means, treatment by iodine, cod-liver oil and tonics is added, I believe marked amendment and cure will oftentimes be obtained when but little hope was entertained from either ere change of climate was obtained. But, although a residence by the sea is often advantageous in the management of cases dependent on this kind of glandular disturbance, still it often happens that a less bracing and inland locality is more suitable. Individual cases must be managed according to circumstances; but, as a rule, I believe a change of climate and residence is often expedient, and even necessary, when medicinal means solely employed have failed. It has already been hinted that this form of glandular affection, when limited to the external lymphatic ganglia, after arriving at a certain stage and becoming permanent, is frequently unaccompanied with any strongly marked evidences of disturbance of the general health. In fact, it is not rare to see individual cases in which not the slightest effect is produced on the system. I have lately been interested in a case of this description, in which the glandular enlargement, especially of the cervical ganglia, has existed for years, and no perceptible effect has been produced on the constitution and vigor of the patient. The case has been under notice for a considerable period, and a large amount of iodine has been taken without having exercised any or little effect on the glandular system. This form of hypertrophy is more permanent when once established than other chronic enlargements; and frequently no perceptible changes are induced by the variations of seasons and temperatures. But, although this condition of hypertrophy is oftentimes comparatively harmless, still it is apt to progress to such an extent as to cause not only inconvenience but danger from mechanical causes. Moreover, fear is to be entertained that any subsequent complication may give rise to still more important lesions; for it has been noticed that more marked scrofulous and true tuberculous mischief is apt to arise, especially if the strumous diathesis be strongly portrayed; and it is on these grounds, if not on others, that such medicinal and general treatment should be adopted as is most likely not only to cure or amend the affection, but to prevent the occurrence of more distressing and dangerous complications.

Local Treatment.—Although local treatment by means of certain applications has been highly extolled by many observant practitioners, still, I believe that a due consideration of the pathological changes that have induced an alteration in the condition of the affected glandular apparatus will at once show that much advantage is not, as a rule, to be hoped for from mere mechanical and therapeutical applications. When such means are, however, employed, either solely or with internally administered medicines, it is customary to adopt the use of those agents which have already been and will presently be more fully considered, viz.: blistering; paintings with iodine, bromine, and their salts in the forms of tinctures, etc; frictions with various powders, ointments and oils; and the constant application of various solutions containing mineral salts, etc. But much harm instead of good may arise by a too pertinacious use of one or more of these various means. I have not unfrequently seen cuticular and cellular mischief occasioned by a too persistent application of iodine; while little or no appreciable advantage has been derived, so far as diminution in the glandular derangement was concerned. When enlargement is dependent on plastic material, doubtless iodine, etc., topically applied, is of some value; but judging from my own experience, I cannot recommend, with any great hope of success, the treatment of this special form of glandular hypertrophy by local discutients, absorptives, etc. Sometimes diminution in size of implicated glands of this nature does ensue from repeated and long continued topical applications of iodine, mercury, etc.; but then, I believe, the general system has become more or less affected through absorption, as I have seen verified on more than one occasion.

Direct surgical treatment is, however, sometimes needed, as it may become expedient to remove one or more hypertrophied glands, which by reason of accummulation in size, occasion not only deformity and inconvenience, but positive danger. Caustics and the knife are the two chief means in the hands of the surgeon to effect reduction or absolute removal. The application of caustics for such purpose has met with some consideration in previous pages, and I have only to reiterate what was then stated. Although a resort to caustics is in general, I consider, not only erroneous, but decidedly bad practice, still there are occasions when no other agent will prove of like advantage. The way in which caustics act when employed as a means of destruction, is by causing inflammation and suppuration of the tissues with which they are in contact. Such changes are more or less rapidly induced, in proportion to the agent employed; while the disturbance generated is in relation to the extent of local destruction and constitutional temperament. I have frequently known very serious results follow this plan of treatment, and I have rarely put it in practice for the removal of this form of glandular mischief.

When it is really expedient and necessary to take away one or more hypertrophied glands, the knife will be found the most certain, least painful, and efficient means at the disposal of the practical surgeon. Care must, however, be taken to select only such cases as are well marked for operative interference. When the gland-tumors are situated in close relation to large arterial and venous trunks and nerves, extra judgment is required in advising and performing an operation; for experience has fully shown that danger is apt to arise therefrom. An example of injudicious interference has been current since the days of Celsus; and there are few surgeons who cannot recall to recollection similar instances of bad practice. When circumstances are favorable, and removal well and carefully executed, success will in general attend direct and judicious surgical interference; for a slight scar will by the majority of patients, especially by those who take pride in the advantages of personal appearance, be deemed of less moment than an unsightly and irreducible gland swelling.—*Brit. Med. Jour.*

